# SARS-CoV-2 Circulation, Guinea, March 2020–July 2021

**DOI:** 10.3201/eid2802.212182

**Published:** 2022-02

**Authors:** Solène Grayo, Cécile Troupin, Moussa Moïse Diagne, Houlou Sagno, Isabelle Ellis, Bakary Doukouré, Amadou Diallo, Jean-Mathieu Bart, Mohamed Lamine Kaba, Benoit Henry, Billy Sivahera Muyisa, Mamadou Saliou Sow, Ndongo Dia, Ousmane Faye, Sakoba Keita, Noël Tordo

**Affiliations:** Institut Pasteur de Guinée, Conakry, Guinea (S. Grayo, C. Troupin, H. Sagno, I. Ellis, B. Doukouré, N. Tordo);; Institut Pasteur de Dakar, Dakar, Senegal (M.M. Diagne, A. Diallo, N. Dia, O. Faye);; Institut de Recherche pour le Développement-Programme National de Lutte contre la Trypanosomiase Humaine Africaine, Montpellier, France (J.-M. Bart);; Camp Militaire Alpha Yaya, Conakry (M.L. Kaba);; Centre Médico-Social de l’Ambassade de France, Conakry (B. Henry);; Centre Hospitalier de Donka (M.S. Sow);; Alliance for International Medical Action, Guinea (B.S. Muyisa);; Agence Nationale de Sécurité Sanitaire, Conakry (S. Keita)

**Keywords:** COVID-19, complete genome sequencing, variants, coronavirus disease, SARS-CoV-2, severe acute respiratory syndrome coronavirus 2, viruses, respiratory infections, zoonoses, West Africa, Guinea

## Abstract

This overview of severe acute respiratory syndrome coronavirus 2 circulation over 1.5 years in Guinea demonstrates that virus clades and variants of interest and concern were progressively introduced, mostly by travellers through Conakry, before spreading through the country. Sequencing is key to following virus evolution and establishing efficient control strategies.

In Guinea, the index coronavirus disease (COVID-19) case-patient identified on March 12, 2020, was an expatriate traveling back from Europe. Immediately, a COVID-19 task force was established by the Agence Nationale de Sécurité Sanitaire; 6 national laboratories were involved in the diagnosis of severe acute respiratory syndrome coronavirus 2 (SARS-CoV-2) infections. As of July 16, 2021, a total of 24,668 confirmed cases (23,571 recovered persons and 188 deaths) have been reported (https://www.anss-guinee.org). The Institut Pasteur de Guinée has contributed to the testing of >25,000 human nasopharyngeal swab samples. Most samples originated in the Conakry area from the Donka University Hospital and the Alpha Yaya Military Hospital, which serve the general population, and from the Health Center of the French Embassy, which serves mostly expatriates or travelers. We selected a panel of 252 (12.26%) SARS-CoV-2–positive samples taken during March 12, 2020–July 16, 2021, for whole-genome sequencing, which was performed at the World Health Organization Collaborative Centre of the Institut Pasteur de Dakar, to examine the evolution of SARS-CoV-2 in Guinea.

From these 252 samples, 226 sequences were generated; we excluded 90 sequences showing >10% missing nucleotides. We analyzed the remaining 136 (54%) sequences by using Nextclade (https://clades.nextstrain.org) and Pangolin software (https://cov-lineages.org). The Guinea sequences are distributed into 7 clades (Appendix Figure): 20A clade (n = 55, 40.44%), 20B clade (n = 31, 22.80%), 20C clade (n = 1, 0.74%), 20D clade (n = 8, 5.88%), 20I clade (20I/B.1.1.7/Alpha; n = 19, 13.97%), 21A clade (21A/B.1.617.2/Delta; n = 16, 11.76%), and 21D clade (21D/B.1.525/Eta; n = 6, 4.41%) (Figure, panel A). The 7 clades are subdivided into subclades. None of these subclades gather sequences from specific prefectures in Guinea, suggesting that SARS-CoV-2 viruses circulating inside the country are related to Conakry cases. At the time of this writing, >21 sublineages of SARS-CoV-2 viruses were circulating in Guinea (Table).

**Figure:**
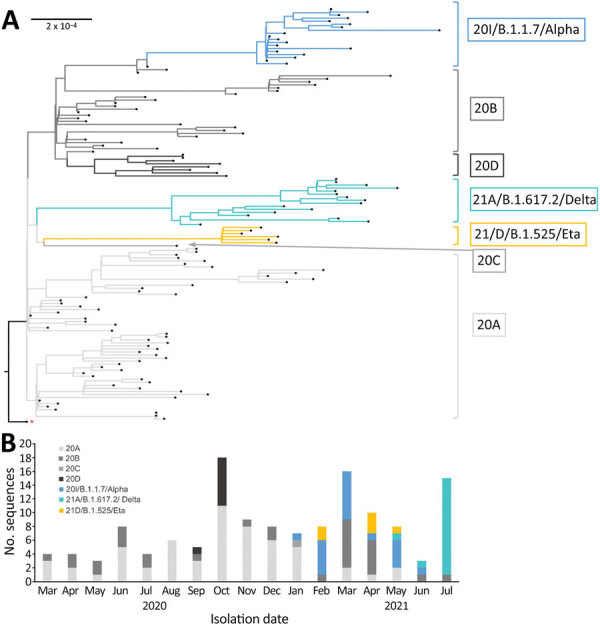
Phylogenetic and temporal descriptions of severe acute respiratory syndrome coronavirus 2 (SARS-CoV-2) sequences from Institut Pasteur de Guinée from samples collected in Guinea during March 12, 2020–July 16, 2021. A) Maximum-likelihood phylogenetic tree of 136 SARS-CoV-2 genomic sequences. The tree was constructed with IQ-tree software by using multiple-genome sequence alignment and Wuhan-Hu-1 strain (GenBank accession no. NC 045512) as outgroup reference sequence, indicated by the red asterisk. Branches and the sequence names are colored according to Nextclade assigned clades: 20A, light gray; 20B, medium gray; 20C, dark gray; 20D, black; 20I/B.1.1.7/Alpha, blue; 21A/B.1.617.2/Delta, azure; 21D/B.1.525/Eta, yellow. Each sequence is highlighted by a black tip. Scale bar indicates the distance corresponding to substitution per site. B) Chronologic distribution of SARS-CoV-2 genomic variants over 17 months in Guinea. The 136 selected sequences are assigned by Nextclade and classified according to sampling date from March 31, 2020, to July 16, 2021. Clades are colored as in panel A.

**Table:** Characteristics of clades and lineages identified among the Institut Pasteur de Guinée SARS-CoV-2 sequences from samples taken in Guinea during March 12, 2020–July 16, 2021*

Clade and lineage	Worldwide				Africa				Guinea†	
	1st described	Location	No. sequences		1st described	Location	No. sequences		1st described	No. sequences
20A										
B.1	2020 Jan	UK	83,632		2020 Mar	RDC	2,816		2020 Mar	43
B.1.36.10	2020 Mar	United States	824		2020 Apr	South Africa	17		2021 Jan	1
B.1.210	2020 Mar	India	403		No	No	0		2020 Oct	1
B.1.243	2020 Mar	United States	13,091		2020 Jun	Kenya	6		2020 Jun	1
B.1.298	2020 Mar	United States	397		No	No	0		2020 Oct	1
B.1.540	2020 Feb	India	2,186		2020 Mar	Gambia, Kenya	134		2020 Jun	2
B.1.622	2021 Jan	Réunion	76		No	No	0		2020 Sep	1
B.1.629	2021 Jan	Belgium	84		Unknown	Guinea	14		2021 Mar	5
20B										
B.1.1	2020 Jan	UK	48,119		2020 Feb	Nigeria	1,361		2020 Mar	16
B.1.1.39	2020 Mar	Switzerland	1,861		No	No	0		2021 Jan	1
B.1.1.142	2020 Mar	Australia	51		No	No	0		2021 Apr	1
B.1.1.236	2020 Feb	UK	1,404		2020 Mar	South Africa	36		2020 Mar	1
B.1.1.316.1‡	2020 Jan	Sierra Leone	10,444		2020 Jan	Sierra Leone	35		2020 Dec	4
B.1.1.317	2020 Feb	Russia	2,435		2020 Jun	Zimbabwe	4		2020 Aug	1
B.1.1.318	2021 Jan	UK	3,350		2021 Jan	Nigeria	360		2021 Feb	6
B.1.1.372	2020 Mar	UK	1,381		2020 May	South Africa	16		2020 Jul	1
20C										
B.1.575	2020 Oct	United States	3,026		2020 Dec	Senegal	12		2021 Jan	1
20D										
B.1.1.1	2020 Mar	UK	3,078		2020 Mar	RDC	169		2020 Sep	8
20I										
B.1.1.7 (Alpha)	2020 Sep	UK	1,045,206		2020 Dec	Ghana	2,047		2021 Jan	19
21A										
B.1.617.2 (Delta)	2020 Nov	India	261,339		2021 Mar	South Africa	1,662		2021 May	16
21D										
B.1.525 (Eta)	2020 Dec	UK, Nigeria	7,752		2020 Dec	Nigeria	581		2021 Jan	6

*Clades and lineages are respectively assigned according to Nextclade definition (https://github.com/nextstrain/ncov/blob/master/docs/src/reference/naming_clades.md) and PANGO lineages list (https://github.com/cov-lineages/pangolin) at the same assignment date (August 14, 2021). The Guinea sequences are distributed in 21 lineages clustered into 7 clades: 20A clade (n = 55, 40.44%) with 8 lineages (B.1, B.1.36.10, B.1.210, B.1.243, B.1.298, B.1.540, B.1.622, and B.1.629), 20B clade (n = 31, 22.80%) with 8 lineages (B.1.1, B.1.1.39, B.1.1.142, B.1.1.236, B.1.1.316.1, B.1.1.317, B.1.1.318, and B.1.1.372), 20C clade (n = 1, 0.74%) with 1 lineage (B.1.575), 20D clade (n = 8, 5.88%) with 1 lineage (B.1.1.1), 20I clade (n = 19, 13.97%) with 1 lineage (B.1.1.7 [Alpha]), 21A clade (n = 16, 11.76%) with 1 lineage (B.1.617.2 [Delta]) and 21D clade (n = 6, 4.41%) with 1 lineage (B.1.525 [Eta]). For each lineage, the first worldwide and African descriptions are provided (date and location), as well as the number of deposited sequences in GISAID (August 16, 2021). RDC, Democratic Republic of the Congo; SARS-CoV-2, severe acute respiratory syndrome coronavirus 2; UK, United Kingdom. †First description and number of sequences in this study. ‡B.1.1.316.1 lineage alias R.1.

During March–August 2020, the sequences were exclusively distributed into 2 clades, 20A and 20B, globally circulating in West and Central Africa (Table; Figure, panel B) (*1*–*3*). Their ancestral position in the maximum-likelihood tree outlines their introduction in Guinea, most likely from Europe as illustrated by the index case. Their circulation has persisted in a nonexclusive manner up to May–July 2021. The 20D clade, sparsely detected in Africa (Table), was observed in Guinea through >2 introductions in September and October 2020, according to the topology of the maximum-likelihood tree (Figure, panel B). Moreover, a single case of 20C clade originating from North America was detected in January 2021 in a person traveling from Haiti (Table; Figure, panel B).

In 2021, new SARS-CoV-2 variants of concern (VOC) and variants of interest, reputed to be more transmissible, emerged in Guinea (*4*). The VOC 20I/B.1.1.7/Alpha variant, which originally emerged in the United Kingdom, was first identified in Guinea in January 2021, increased in incidence up to March 2021, and then decreased from April to June 2021, corresponding to the dynamic described in Africa (Figure, panel B) (*1**–**3**,**5*; E.A. Ozer et al., unpub. data, https://www.medrxiv.org/content/10.1101/2021.04.09.21255206v3). The variant of interest 21D/B.1.525/Eta was identified in Guinea and other countries in Central and West Africa in February–May 2021 (Table) (*5*; E.A. Ozer et al., unpub. data). The topology of the Guinea maximum-likelihood tree with only one subclade of this variant suggests a unique introduction in this study. Finally, the 21A/B.1.617.2/Delta VOC was first detected in May 2021 in Guinea (Figure, panel B). By July, it had become dominant; >90% of the sequenced viruses by Institut Pasteur de Guinée demonstrated the same dynamics observed during May–August 2021 in Africa (*6*). The maximum-likelihood tree suggests >2 main introductions of this variant in Guinea.

In summary, although only 20A and 20B clades circulated in Guinea for the first 6 months of the pandemic (March–August 2020), the reopening of borders and commercial flights have progressively enabled the introduction of variants from surrounding parts of Africa (21D/B.1.525/Eta) and globally (20I/B.1.1.7/Alpha and 21A/B.1.617.2/Delta) several months after their original detection (Table). Although the 20I/B.1.1.7/Alpha and 21A/B.1.617.2/Delta variants have spread successfully in the population, the 21D/B.1.525/Eta variant has only occasionally been detected. We did not detect other variants previously found in Africa, such as the 20H/B.1.351/Beta variant (which populated 50% of sequences in Africa during January–May 2021) and variants from the sublineage A, including the A.23.1 lineage from East Africa and the A.27 lineage of uncertain origin, in this study (*1*–*3*,*5*; E.A. Anoh et al., unpub. data, https://www.medrxiv.org/content/10.1101/2021.05.06.21256282v1).

This overview of the circulation of SARS-CoV-2 viruses in Guinea furthers the examination of infectious diseases control strategies in Africa, which faces vaccination implementation delay (*7*). Beside classical quantitative reverse transcription PCR diagnostic testing, strengthening of the sequencing capacity is the cornerstone of tracking and fighting the emergence of SARS-CoV-2 variants in real time (*8*). Making countries autonomous in sequencing is the next challenge in fighting COVID-19, as well as other emerging zoonoses, in Africa.

Appendix Additional information about SARS-CoV-2 circulation, Guinea, March 2020–July 2021
