# Supplementary material for: SARS-CoV-2 Circulation, Guinea, March 2020–July 2021
Source: Emerg Infect Dis. 2022 Feb;28(2):457–60. doi: 10.3201/eid2802.212182 (PMC8798712; doi:10.3201/eid2802.212182)
Supplement: Appendix — Additional information about SARS-CoV-2 circulation, Guinea, March 2020–July 2021 [file 21-2182-Techapp-s1.pdf]

Article DOI: https://doi.org/10.3201/eid2802.212182

SARS-CoV-2 Circulation, Guinea, March 2020–July 2021

Appendix

Materials and Methods

Samples Origin

Institut Pasteur de Guinée (IPGui) is in charge of severe acute respiratory syndrome coronavirus 2 (SARS-CoV-2) molecular diagnostics at 3 sites in Conakry, Guinea, including 2 Centre de Traitement des Épidémies (CTEPI): the CTEPI of the Donka University Hospital (serving the general public up to severe cases requiring oxygen supplementation), the CTEPI of the Alpha Yaya Military Hospital (for members of the military and their families), and the sociomedical center of the French Ambassy (for expatriates [suspected and control patients]).

Coronavirus Disease Diagnosis

Viral RNA isolation

Nucleic acid extraction was carried out by using ID Gene Mag Fast Extraction Kit and IDEAL 32 extraction robot (ID-Vet, https://www.id-vet.com) according to the manufacturer's instructions from 140 µL of nasopharyngeal swab specimens. The extracted RNA was eluted in 80 µL of elution buffer. Positive and negative controls are included in each extraction run.

Quantitative Reverse Transcription PCR

SARS-CoV-2 genome detection was performed on extracted RNA (no freezing) using the 2019-nCoV Nucleic Acid Diagnostic Kit (Sansure Biotech, https://www.sansureglobal.com) under US Food and Drug Administration Emergency Use Authorization. This quantitative reverse transcription PCR kit targets 2 SARS-CoV-2 genes (nucleocapsid N and polymerase ORF1ab) and the human RNase P gene, as Internal Control (IC).

Positive and negative controls are included in each amplification run performed in a LightCycler Roche (Roche, https://www.roche.com) machine. Left extracted RNA is immediately stored at −80°C in the IPGui Biobank.

Sample Selection for Sequencing

During March, 12 2020-July 16, 2021, 22,975 human nasopharyngeal swab samples were stored at −80°C at IPGui Biobank. Among the 2,055 positive patients, 252 (12.26%) were selected with the following characteristics: a) having a cycle threshold value <30 to improve sequence quality; b) following the dynamics of the epidemic with ≥10 samples per month with higher numbers from 2021 and the arrival of the variant of concern and variant of interest in Africa; c) including samples from outside Conakry (n = 47, 39%). Aliquots of 500 μL of nasopharyngeal swabs from the 252 selected samples were shipped at 4°C to Institut Pasteur de Dakar to perform Illumima sequencing.

Next-Generation Sequencing

Next-generation sequencing was performed at Institut Pasteur de Dakar. Briefly, viral RNA was extracted using the QIAamp viral RNA minikit (QIAGEN, https://www.qiagen.com) following manufacturer recommendations. SARS-CoV-2 genomes were generated by an amplicon-based approach using either the Illumina DNA Prep, (M) Tagmentation (96 Samples) kit or the US Food and Drug Administration–approved Illumina COVIDSeq kit (Illumina, https://www.illumina.com), depending on availability.

The first protocol consisted of a reverse complement step on the RNA extracts for cDNA synthesis before performing generation of tiled amplicons by reverse transcription PCR (RT-PCR) made with the ARTIC primers version 3 of the nCoV-2019 as previously described (https://www.protocols.io/view/ncov-2019-sequencing-protocol-v3-locost-bh42j8ye). The PCR products were purified and DNA was quantified with a Qubit 3 fluorometer (ThermoFisher Scientific, https://www.thermofisher.com). DNA products (multiplex PCR pools A and B) were pooled in equal concentrations and libraries were generated by using the Illumina DNA Prep, (M) Tagmentation (96 Samples) according to the manufacturer’s specifications. Whole-genome sequencing was performed with paired-end reads by using the Illumina MiSeq reagent kit v3 (150 cycles) on an Illumina MiSeq instrument.

The Illumina COVIDSeq protocol is a modified version of the previous one, with 98 amplicons designed to amplify SARS-CoV-2–specific sequences, combined with proven Illumina sequencing technology (*1*). Final libraries were loaded in a NextSeq550 sequencer according to the manufacturer’s recommendations.

Genome Assembly

Genome consensus was generated using the EDGE COVID-19 pipeline (C.-C. Lo, unpub. data, https://arxiv.org/abs/2006.08058), a tailored bioinformatics platform based on the fully open-source EDGE bioinformatics software (*2*). Preprocessing (data quality control) was performed using FastQCs. This analysis included trimming low-quality regions of reads and filtering reads that either failed a quality threshold or minimum length. We used the align_trim.py (from ARTIC) to soft clip primer regions from the alignment file (BAM) based on the position of primers in the reference genome (using primer BED file).

Reads were aligned to the original reference genome (NC_045512.2) after removing the PolyA-tail from the 3′ end (33 nt). BWA mem was used as the default aligner, which was then automatically followed by variant calling and generation of a consensus sequence.

Variant calling used bcftools mpileup command to convert the aligned BAM file into genomic positions and call genotypes, reduced the list of sites to those found to be variants by passing this file into bcftools call. The variant calls were further filtered by vcfutils.pl (from samtools). The consensus workflow used a maximum of 8000× depth coverage reads for computational efficiency. Various other parameters were defaulted including a minimum depth coverage (5×) of support of a variant site coverage per base (otherwise the consensus will be "N"), base quality (20), alternate base threshold (0.5) to support an alternative for the consensus to be changed, indels threshold (0.5) to support an INDEL for the consensus to be changed, and minimum mapping quality of 60. All the sequences generated in this study have been submitted to GISAID

Clade and Lineage Assignation

Nextclade tool (version 16.0) (https://clades.nextstrain.org) has been used to identify mutations (n = 136) compared with the SARS-CoV-2 reference sequence (Wuhan-Hu-1; NC_045512). The Nexclade tool uses these mutations to assign the sequences to specific clades and to place them on a reference phylogenetic tree with a subset of all sequences available in GISAID.

Phylogenetic Analysis

A multiple sequence alignment of the 136 new SARS-CoV-2 genomes from Guinea combined with the SARS-CoV-2 reference sequence NC_045512 from Genbank was constructed using MUSCLE (http://www.ebi.ac.uk/Tools/msa/muscle) (*3*) with default parameters implemented in Galaxy (*4–6*) (https://www.research.pasteur.fr/en/tool/pasteur-galaxy-platform). A phylogenetic tree was then estimated by using W-IQ-TREE, a web

interface and server for IQ-TREE that is a phylogenetic software for maximum likelihood (ML) analysis (*7*) (http://iqtree.cibiv.univie.ac.at/). The best-fit model for nucleotide substitution according to the Bayesian Information Criterion (BIC) determined by Model Finder (*8*) was the general time-reversible model with proportion of invariable sites plus gamma-distributed rate heterogeneity (GTR+I+G4). This project was approved by the Comité National d'Éthique pour la Recherche en Santé (CNERS) with reference number 109/CNERS/20 (28/08/2020) 152/CNERS/20 (05/011/2020)

References

1. Bhoyar RC, Jain A, Sehgal P, Divakar MK, Sharma D, Imran M, et al. High throughput detection and genetic epidemiology of SARS-CoV-2 using COVIDSeq next-generation sequencing. PLoS One. 2021;16:e0247115. PubMed https://doi.org/10.1371/journal.pone.0247115

2. Li PE, Lo CC, Anderson JJ, Davenport KW, Bishop-Lilly KA, Xu Y, et al. Enabling the democratization of the genomics revolution with a fully integrated web-based bioinformatics platform. Nucleic Acids Res. 2017;45:67–80. PubMed https://doi.org/10.1093/nar/gkw1027

3. Edgar RC. MUSCLE: a multiple sequence alignment method with reduced time and space complexity. BMC Bioinformatics. 2004;5:113. PubMed https://doi.org/10.1186/1471-2105-5-113

4. Goecks J, Nekrutenko A, Taylor J, Galaxy Team T; Galaxy Team. Galaxy: a comprehensive approach for supporting accessible, reproducible, and transparent computational research in the life sciences. Genome Biol. 2010;11:R86. PubMed https://doi.org/10.1186/gb-2010-11-8-r86

5. Blankenberg D, Von Kuster G, Coraor N, Ananda G, Lazarus R, Mangan M, et al. Galaxy: a web-based genome analysis tool for experimentalists. Curr Protoc Mol Biol. 2010; Chapter 19:Unit 19.10.1–21.

6. Giardine B, Riemer C, Hardison RC, Burhans R, Elnitski L, Shah P, et al. Galaxy: a platform for interactive large-scale genome analysis. Genome Res. 2005;15:1451–5. PubMed https://doi.org/10.1101/gr.4086505

7. Trifinopoulos J, Nguyen L-T, von Haeseler A, Minh BQ. W-IQ-TREE: a fast online phylogenetic tool for maximum likelihood analysis. Nucleic Acids Res. 2016;44:W232–5. PubMed https://doi.org/10.1093/nar/gkw256

8. Kalyaanamoorthy S, Minh BQ, Wong TKF, von Haeseler A, Jermiin LS. ModelFinder: fast model selection for accurate phylogenetic estimates. Nat Methods. 2017;14:587–9. PubMed https://doi.org/10.1038/nmeth.4285

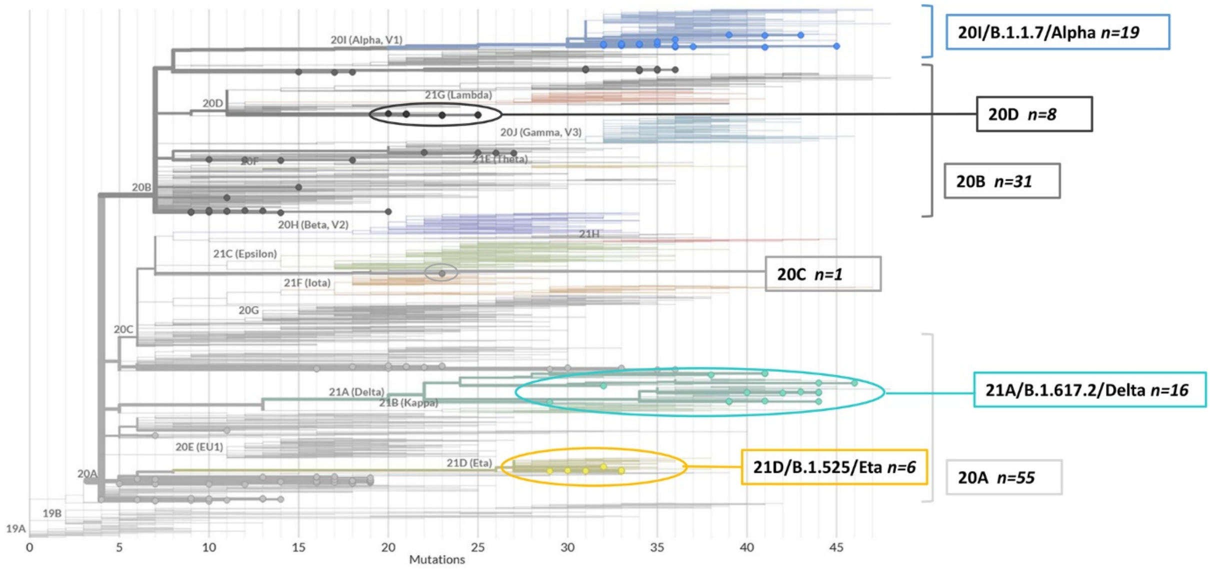

Appendix Figure. Institut Pasteur de Guinée severe acute respiratory syndrome coronavirus 2 sequences' position on the global Nextclade phylogenetic tree. The tree is generated from Nextclade online software (https://clades.nextstrain.org/) and rooted with the Wuhan sequence reference (NC 045512 Wuhan-Hu-1, WIV04-reference). The x-axis labels the mutation number compared with the Wuhan sequence. Each Guinea sequence is highlighted by a bold circle to facilitate its position in the global tree. The 136 sequences from Institut Pasteur de Guinée Biobank are distributed into 7 clades: 20A, light gray; 20B, medium gray; 20C, dark gray; 20D, black; 20I/B.1.1.7/Alpha, blue; 21A/B.617.2/Delta, turquoise; 21D/B.1.525/Eta, yellow.
